# Effect of smoking on lung function, respiratory symptoms and respiratory diseases amongst HIV-positive subjects: a cross-sectional study

**DOI:** 10.1186/1742-6405-7-6

**Published:** 2010-03-19

**Authors:** Qu Cui, Sue Carruthers, Andrew McIvor, Fiona Smaill, Lehana Thabane, Marek Smieja

**Affiliations:** 1 Department of Clinical Epidemiology and Biostatistics, McMaster University, 1200 Main Street West, Hamilton, ON L8N 3Z5, Canada; 2 St Joseph's Hospital, Hamilton, ON, Canada; 3 Department of Medicine, McMaster University, Hamilton, ON, Canada; 4 Department of Pathology and Molecular Medicine, McMaster University, Hamilton, ON, Canada

## Abstract

**Background:**

Smoking prevalence in human immunodeficiency virus (HIV) positive subjects is about three times of that in the general population. However, whether the extremely high smoking prevalence in HIV-positive subjects affects their lung function is unclear, particularly whether smoking decreases lung function more in HIV-positive subjects, compared to the general population. We conducted this study to determine the association between smoking and lung function, respiratory symptoms and diseases amongst HIV-positive subjects.

**Results:**

Of 120 enrolled HIV-positive subjects, 119 had an acceptable spirogram. Ninety-four (79%) subjects were men, and 96 (81%) were white. Mean (standard deviation [SD]) age was 43.4 (8.4) years. Mean (SD) of forced expiratory volume in one second (FEV_1_) percent of age, gender, race and height predicted value (%FEV_1_) was 93.1% (15.7%). Seventy-five (63%) subjects had smoked 24.0 (18.0) pack-years. For every ten pack-years of smoking increment, %FEV_1 _decreased by 2.1% (95% confidence interval [CI]: -3.6%, -0.6%), after controlling for gender, race and restrictive lung function (R^2 ^= 0.210). The loss of %FEV_1 _in our subjects was comparable to the general population. Compared to non-smokers, current smokers had higher odds of cough, sputum or breathlessness, after adjusting for highly active anti-retroviral therapy (HAART) use, odds ratio OR = 4.9 (95% CI: 2.0, 11.8). However respiratory symptom presence was similar between non-smokers and former smokers, OR = 1.0 (95% CI: 0.3, 2.8). All four cases of COPD (chronic obstructive pulmonary disease) had smoked. Four of ten cases of restrictive lung disease had smoked (p = 0.170), and three of five asthmatic subjects had smoked (p = 1.000).

**Conclusions:**

Cumulative cigarette consumption was associated with worse lung function; however the loss of %FEV_1 _did not accelerate in HIV-positive population compared to the general population. Current smokers had higher odds of respiratory symptoms than non-smokers, while former smokers had the same odds of respiratory symptoms as non-smokers. Cigarette consumption was likely associated with more COPD cases in HIV-positive population; however more participants and longer follow up would be needed to estimate the effect of smoking on COPD development. Effective smoking cessation strategies are required for HIV-positive subjects.

## Background

In the developed world, mortality from HIV/AIDS has decreased significantly since the introduction of highly active anti-retroviral therapy (HAART) in 1996 [1]. Consequently, people are living with HIV/AIDS longer than ever. In this context, chronic diseases, whether HIV/AIDS related or not, are increasingly of concern amongst the HIV-positive population, and for clinicians caring for them.

Prior to 2001, annual smoking prevalence in the Ontario Cohort Study (OCS) of HIV-positive adult subjects was more than 70%, and steadily decreased to 58% in 2007 (data unpublished), which was constantly about three times higher than that in the Ontario general population from 1999 to 2007 [2]. A smoking prevalence of 60% or more in HIV-positive subjects has been reported in other studies [3-7]. Therefore, smoking-related outcomes, such as lung function problems, respiratory symptoms and lung diseases, are likely to increase in this population.

We hypothesized that HIV infection would accelerate smoking-related respiratory symptoms and diseases. Studies have shown that HIV-positive subjects were more likely to have respiratory symptoms and diseases compared to their HIV negative counterparts [3,8,9]. Moreover, some studies showed among HIV-positive subjects, smokers were more likely to have respiratory problems [8,10]. Other studies found that HIV-positive subjects had similar lung function compared to their HIV negative counterparts [11,12], and they had similar changes of lung function over time [13,14]. Although in the general population, the effects of cigarette smoking on lung function has been well demonstrated in the general population, using different measurements of smoking and lung function [15-21], no study has been done to address whether cigarette smoking affects lung function in a similar way in HIV-positive population.

Hence, the literature is unclear on the effects of smoking on lung function in HIV-positive subjects, particularly whether lung function decline would be greater in HIV-positive subjects compared to the general population. The primary objective of this study was to determine the association between smoking and lung function amongst HIV-positive subjects. The secondary objective was to examine the association between smoking and respiratory symptoms and diseases amongst HIV-positive subjects.

## Methods

### Study design, setting and participants

This was a cross-sectional study. The study protocol was approved by the Research Ethics Board (REB) at Hamilton Health Sciences/McMaster University. Consecutive consenting HIV-positive subjects attending the regional HIV clinic (Special Immunology Services [SIS] clinic) at McMaster University, aged 18 years or more were eligible to take part in the study.

### Study description

Our study respiratory technologist approached potentially eligible subjects attending regularly scheduled clinical visits at the SIS clinic. Participants provided signed informed consent, filled out a questionnaire, and underwent spirometry testing. The questionnaire contained information on demography, respiratory symptoms, and history of respiratory diseases, cigarette smoking and other drug uses. Spirometry testing followed the standardization of spirometry testing recommended by the American Thoracic Society (ATS) and European Respiratory Society (ERS) [22-24]. We used a VIASYS JAEGER FlowScreen V2.1.1 (Hoechberg, Germany). All subjects who had forced expiratory volume in one second (FEV_1_) percent of age, gender, race and height predicted value (%FEV_1_) less than 90% were given two puffs of salbutamol (Ventolin, a short acting β-agonist) and repeated spirometry to assess the change in FEV_1_. Medical information such as CD4 T-lymphocyte count, HIV viral load, date of HIV diagnosis and antiretroviral medication was abstracted from the medical chart. Information on history of respiratory diseases was abstracted from the medical chart if information was absent in the questionnaire.

### Measurements

Each subject was self-classified as a non-smoker, ex-smoker or current smoker. Cumulative exposure to cigarette smoking was measured by pack-years, which was calculated by multiplying the number of packs of cigarette smoked per day and the number of years of smoking. Marijuana use was similarly measured as never, former and current use. Marijuana consumption was measured by the number of times of use per day and the number of years of use. The primary outcome of lung function was measured as FEV_1_, %FEV_1_, forced vital capacity (FVC) and FVC percent of age, gender, race and height predicted value (%FVC). All measurements were automatically printed by the VIASYS JAEGER FlowScreen. Per cent FEV_1 _and %FVC were calculated by dividing the measured FEV_1 _and FVC by their age, gender, race and height predicted values, which were also automatically printed by FlowScreen. Respiratory symptoms included cough, sputum and breathlessness. Cough was described as current cough, productive cough and nocturnal cough. Sputum was measured at 5 levels: no sputum, 1 tea spoon, 1 table spoon, 2 table spoon and 1/2 cup in 24 hours. We used the Medical Research Council (MRC) dyspnea scale to measure breathlessness. Grade 3 or more (breathlessness walking on the level) was considered having breathlessness [25].

The diagnoses of obstructive and restrictive lung diseases could be interpreted by the spirogram, however we diagnosed these diseases based on our calculation and the GOLD (Global initiative for chronic Obstructive Lung Disease) guidelines. The diagnosis of obstructive lung function was defined as pre-salbutamol FEV_1_/FVC < 70% without post-salbutamol values. The diagnosis of COPD was defined by post-bronchodilator FEV_1_/FVC < 70%, and COPD level was classified based on %FEV_1_. A COPD case with %FEV_1 _≥ 80% was classified as mild, 30% ≤ %FEV_1 _< 80% as moderate and %FEV_1 _< 30% as severe [26]. Subjects who did not undergo post-salbutamol testing could not be diagnosed as COPD by definition. For subjects whose post-salbutamol FEV_1_/FVC was between 66.5% and 73.5%, the diagnosis was made by the committee's judgment, taking account of the pre-salbutamol FEV_1_/FVC value and clinical symptoms of cough, sputum and breathlessness. The diagnosis of restrictive lung function was defined as FEV_1_/FVC ≥ 70% and %FVC < 80%, either before or after salbutamol inhalation [27,28]. Asthma was defined as reversible FEV_1_, which improved more than 12% and 200 ml after salbutamol inhalation [26]. A history of asthma was defined as previous diagnosed or treated asthma. Normal lung function was defined as FEV_1_/FVC ≥ 70%, %FEV_1 _≥ 80% and %FVC ≥ 80% accordingly, by both pre- and post-salbutamol tests. Pre-salbutamol values were used to classify normal lung function if post-salbutamol test was not done.

### Statistical analysis

Continuous variables were reported as mean (standard deviation [SD]) if they were normal distributed or reported as median (first quartile [Q_1_], third quartile [Q_3_]) if they were not normal distributed. Normality was visually tested by P-P plots. Categorical variables were reported as count (percent). Analysis of variance (ANOVA) was used to compare continuous variables among different groups and χ^2 ^test was used for categorical variables. Fisher's exact test was adopted if the number of cases in any cell is less than 10. Multiple regression method was used to adjust for possible confounders further. Multiple linear regression was used to model %FEV_1_. Multiple logistic regression model was used for lung function, respiratory symptoms, respiratory diseases and subject classification. We used the criterion of α = 0.20 in uni-variate regression analysis to decide whether or not to select appropriate variables into a multivariable regression model. Possible interaction between independent variables was tested. The criterion for statistical significance for multivariable analysis was set at α = 0.05. All p-values were reported to three digital places with those less than 0.001 were reported as p < 0.001. All analyses were performed using SPSS 15 (Chicago, IL).

In the multiple linear regression model, the dependent variable was %FEV_1 _before salbutamol. The selected predictor was pack-years of smoking or smoking status, depending on which variable had the smaller p value in uni-variate analysis. Restrictive lung function was co-variable related to %FEV_1_. Productive cough and age were potential confounders based on the previous literature [17,19,20]. We examined gender and race as they were common confounders, although previous studies were inconclusive [18,19]. In addition, current CD4 T-lymphocytes count, current viral load, current antiretroviral treatment and marijuana use were examined a priori as potential confounders as well. Potential interactions between independents were tested. The results were reported as estimates of model coefficients (95% confidence interval [CI]) and associated p-values. Results for all subjects and for smokers (including former and current smokers) were presented when pack-years of smoking was the predictor variable. We examined the residuals to assess model assumptions and goodness-of-fit (reported using R^2^).

In the multiple logistic regression model, the dependent variable was lung function (normal/abnormal), respiratory symptom (yes/no), respiratory disease (yes/no) and subject classification (normal lung function and no symptom/abnormal lung function or respiratory symptom) in each analysis respectively, and the predictor was smoking status. We considered the same potential confounders as we did in multiple linear regression analysis. We selected no more than one independent variable for each ten cases, which was considered to be the lesser number of the outcome group. If the lesser number of the outcome group was less than 10, logistic regression analysis was not conducted. The results were reported as estimates of odds ratio (OR) (95% CI) and their p-values. Nagelkerke R square was reported to assess the goodness-of-fit of logistic regression model.

## Results

### Demographic and baseline information

We recruited 120 consecutive consenting HIV positive subjects, of whom 119 had an acceptable spirogram. Demographic and baseline information are listed in Table 1. Ninety-four (79%) subjects were men (one trans-gendered individual was classified as a woman). Ninety-six (81%) subjects were white, including 83 (88%) men and 13 (52%) women (p < 0.001). Mean (standard deviation [SD]) age was 43.4 (8.4) years. Men were 5.4 years older than women (p = 0.004). Mean (SD) number of years of living with HIV was 9.0 (6.6) years. One hundred (84%) HIV-positive subjects were on antiretroviral treatment at the time of study. Mean (SD) current CD4 T-lymphocytes count was 484 (274) cells/mm^3^, and 102 (86%) of subjects had current CD4 count of 200 cells/mm^3 ^or more. Seventy-three (61%) subjects had current undetectable viral load. Amongst those with detectable viral load, median (Q_1_, Q_3_) viral load was 907 (193, 28630), and mean (SD) of log viral load was 3.38 (1.26). No gender or race difference was found in terms of current CD4 T-lymphocytes count or HIV viral load.

**Table 1:** Demographic and baseline information by gender and smoking status (n = 119)

	Gender		Smoking status			
			
	Male (n = 94)	Female (n = 25)	None (n = 44)	Former (n = 23)	Current (n = 52)	Total (n = 119)
Male, n (%)	-	-	30 (68)	21 (91)	43 (83)	94 (79)
White, n (%)	83 (88)	13 (52)	28 (64)	19 (83)	49 (94)	96 (81)^***##^
Age (years), mean (SD)	44.5 (8.3)	39.2 (7.7)	42.7 (7.7)	46.0 (8.8)	42.8 (8.8)	43.4 (8.4) **
Years of living with HIV, mean (SD)	9.6 (6.7)	6.9 (6.0)	7.3 (5.8)	10.6 (6.9)	9.7 (7.0)	9.0 (6.6)
On HAART, n (%)	86 (92)	14 (56)	35 (80)	22 (96)	43 (83)	100 (84) ***
CD4 (cells/mm^3^), mean (SD)	478 (264)	505 (312)	510 (261)	402 (252)	498 (291)	484 (274)
Undetectable viral load, n (%)	62 (66)	11 (44)	25 (57)	17 (74)	31 (60)	73 (61)
Pack-years in smokers^1^, mean (SD)	24.0 (17.6)	24.0 (20.7)	-	16.8 (13.9)	27.2 (18.7)	24.0 (18.0)^#^
Marijuana use						^###^
None, n (%)	42 (45)	18 (72)	34 (77)	9 (39)	17 (33)	60 (50)
Former, n (%)	24 (26)	5 (20)	7 (16)	8 (35)	14 (27)	29 (24)
Current, n (%)	28 (30)	2 (8)	3 (7)	6 (26)	21 (40)	30 (25)
FEV_1 _before salbutamol (litres), mean (SD)	3.8 (0.7)	2.7 (0.5)	3.48 (0.87)	3.46 (0.81)	3.62 (0.79)	3.5 (0.8)***
%FEV_1 _before salbutamol (%), mean (SD)	94.7 (15.8)	86.9 (14.1)	93.6 (14.0)	91.1 (18.3)	93.5 (16.1)	93.1 (15.7)*
FVC before salbutamol (litres), mean (SD)	4.9 (0.8)	3.3 (0.7)	4.26 (1.09)	4.58 (0.89)	4.76 (0.95)	4.5 (1.0)***
%FVC before salbutamol (%), mean (SD)	96.2 (12.5)	88.2 (16.7)	91.4 (13.7)	94.6 (11.7)	97.1 (14.4)	94.5 (13.8)**
Abnormal lung function^2^, n (%)	16 (17)	8 (32)	8 (18%)	4 (17%)	12 (23%)	24 (20)
Asthma history, n (%)	6 (7)	7 (29)	3 (7)	3 (13)	7 (14)	13 (11)**
Asthmatic by spirometry^3^, n (%)	3 (3)	2 (8)	2 (5)	1 (4)	2 (4)	5 (4)
Cough, n (%)	49 (52)	12 (48)	16 (36)	8 (35)	37 (71)	61 (51)^##^
Sputum, n (%)	41 (44)	10 (40)	11 (25)	8 (35)	32 (63)	51 (43)^##^
Breathlessness, n (%)	6 (6)	2 (8)	0	1 (4)	7 (14)	8 (7)^#^
Any respiratory symptom^4^, n (%)	51 (54)	12 (48)	16 (36)	9 (39)	38 (73)	63 (53)^##^
COPD^5^, n (%)	4 (4)	0	0	2 (9%)	2 (4%)	4 (3)
Restrictive lung diseases^6^, n (%)	3 (3)	7 (28)	6 (14%)	1 (4%)	3 (6%)	10 (8)**
Abnormal lung function^2 ^or symptomatic^4^, n (%)	17 (68)	54 (57)	20 (45)	11 (48)	40 (77)	71 (60)^##^

P value was obtained by Analysis of variance (ANOVA) for continuous variables listed as mean (SD) and was obtained by χ^2 ^test for categorical variables listed as n (%). Fisher's exact test was adopted if the number of cases in any cell was less than 10.

- Not applicable.

* p < 0.05 by gender. ** p < 0.01 by gender. *** p < 0.001 by gender.

^# ^p < 0.05 by smoking status. ^## ^p < 0.01 by smoking status. ^### ^p < 0.001 by smoking status.

^1^Pack-year of smoking was calculated by multiplying the number of packs of cigarette smoked per day and the number of years of smoking. ^2^Abnormal lung function was defined as either FEV_1_/FVC < 70% or %FEV_1 _<80% or %FVC < 80%, either pre- or post-salbutamol test. ^3^Asthmatic by spirometry was defined as reversible FEV_1_, which improved more than 12% and 200 ml after salbutamol inhalation. ^4^Any respiratory symptom was defined as having cough, sputum or breathlessness. ^5^COPD referred to chronic obstructive pulmonary disease, was defined as post-salbutamol FEV_1_/FVC < 70%. ^6^Restrictive lung function was defined as FEV_1_/FVC ≥ 70% and %FVC < 80%, either before or after salbutamol inhalation.

### Smoking status and marijuana use

Forty-four (37%) subjects never smoked cigarettes, of whom 3 subjects currently used marijuana at the time of survey. Twenty-three (19%) subjects had formerly smoked, of whom 6 subjects currently used marijuana. Fifty-two (44%) subjects currently smoked, of whom 21 subjects currently used marijuana. Males accounted for 68% in non-smokers and 85% in smokers respectively (p = 0.036). On average smokers had smoked 24.0 (18.0) pack-years. Mean (SD) pack-years of smoking was 16.8 (13.9) for former smokers and 27.2 (18.7) for current smokers respectively (p = 0.020).

Sixty (50%) subjects never used marijuana. Twenty-nine (24%) subjects formerly used marijuana, of whom only 7 (24%) subjects used once or more per day, with mean (SD) year of use of 8.7 (4.5) years. Thirty (25%) subjects were currently using marijuana at the time of survey, of whom 26 (87%) subjects used once or more per day, with mean (SD) year of use of 18.3 (9.5) years. Current users used marijuana more frequently (p < 0.001) and for a longer time (p < 0.001) than former users.

### Association between lung function and smoking

Lung function by smoking status and gender was summarized in Table 1. Mean (SD) of FEV_1 _before salbutamol was 3.5 (0.8) litres. Mean (SD) of %FEV_1 _before salbutamol was 93.1% (15.7%). Mean FVC before salbutamol was 4.5 (1.0) litres. Forty-six (39%) HIV-positive subjects had %FEV_1 _< 90% and 27 (59%) of them underwent post salbutamol spirometry test. Mean improvement was 143 (193) ml for FEV_1 _and 79 (263) ml for FVC respectively.

According to our preset criterion of α = 0.2, four variables from uni-variate regression were selected to build the multiple linear regression model: pack-years, gender, race and restrictive lung function. For every ten pack-years of smoking increment, %FEV_1 _significantly decreased by 2.1% (95% CI: -3.6%, -0.6%), after controlling for gender, race and restrictive lung diseases (p = 0.006). Moreover white subjects had 8.8% (95% CI: 1.2%, 16.3%) higher %FEV_1 _than non-white, after controlling for pack-years, gender and restrictive lung diseases (p = 0.023). Gender did not affect %FEV_1 _significantly (p = 0.640). No interaction between independents was found. The point estimate of β coefficient of -2.0% (95% CI: -4.2%, 0.2%) was similar when non-smokers were excluded, with wider 95% CI (p = 0.077). The point estimate of pack-years did not change when the association was not adjusted for gender. Coefficients and 95% CIs of each variable versus %FEV_1 _in different populations were summarized in Table 2.

**Table 2:** β Coefficients and 95% CIs of each variable versus %FEV_1 _by model and population

	In all the subjects		In smokers	
	
	Model 1^#^	Model 2^##^	Model 1^#^	Model 2^##^
Per 10 pack-years	-0.021 (-0.036, -0.006)**	-0.021 (-0.036, -0.006)**	-0.020 (-0.042, 0.002)	-0.020 (-0.042, 0.002)
Male	0.017 (-0.054, 0.088)	Not assessed	-0.034 (-0.144, 0.077)	Not assessed
White	0.088 (0.012, 0.163)*	0.093 (0.020, 0.165)*	0.050 (-0.086, 0.187)	0.052 (-0.083, 0.188)
Restrictive lung diseases	-0.167 (-0.270, -0.065)**	-0.174 (-0.272, -0.077)**	-0.133 (-0.309, 0.042)	-0.121 (-0.290, 0.049)

All the results were from the multiple linear regression analysis, in which the dependent variable was %FEV_1. _Pack-year of smoking was calculated by multiplying the number of packs of cigarette smoked per day and the number of years of smoking. Restrictive lung function was defined as FEV_1_/FVC ≥ 70% and %FVC < 80%, either before or after salbutamol inhalation.

^# ^In model 1, the association between %FEV_1 _and pack-years of smoking was adjusted for by gender, race and restrictive lung diseases. R^2 ^= 0.210 in all the subjects and R^2 ^= 0.084 in smokers.

^## ^In model 2, the association between %FEV_1 _and pack-years of smoking was adjusted for by race and restrictive lung diseases. R^2 ^= 0.209 in all the subjects and R^2 ^= 0.080 in smokers.

* p < 0.05 for β coefficient. **p < 0.01 for β coefficient.

Among 24 (20%) subjects who had abnormal lung function, there were 8 non-smokers, 4 former smokers and 12 current smokers respectively (p = 0.782) (Table 1). According to preset criterion of α = 0.2, smoking status was not selected into multiple logistic regression model for abnormal lung function.

### Association between respiratory symptoms and smoking

#### Cough

Sixty-one (51%) subjects coughed, including 16 (36%) non-smokers, 8 (35%) former smokers and 37 (71%) current smokers (p = 0.001) (Table 1). Compared to non-smokers, current smokers had higher odds of cough, OR = 4.3 (95% CI: 1.5, 12.0) after controlling for marijuana use, race, current HAART status and current viral load (p = 0.005). For former smokers the OR of 0.8 (95% CI: 0.3, 2.6) was not statistically significant compared to non-smokers (p = 0.753). No interaction was found. Moreover, subjects who were on HAART had higher odds of cough (OR = 5.5, 95% CI: 1.4, 21.5, p = 0.014), and subjects who had suppressed viral load had lower odds of cough (OR = 0.3, 95% CI: 0.1, 0.9, p = 0.025). The results are presented in Table 3.

**Table 3:** ORs and 95% CIs of each variable versus respiratory symptoms, respiratory diseases and subject classification

	Cough^a^	Sputum^b^	Any respiratory symptom^c^	Normal lung function without symptom^d^
Former smoker	0.8 (0.3, 2.6)	1.7 (0.5, 5.3)	1.0 (0.3, 2.8)	0.9 (0.3, 2.5)
Current smoker	4.3 (1.5, 12.0)**	5.0 (1.9, 13.3) **	4.9 (2.0, 11.8) ***	0.3 (0.1, 0.6) **
Former marijuana user	0.7 (0.3, 2.1)	0.6 (0.2, 1.7)	-	-
Current marijuana user	1.5 (0.5, 4.6)	1.3 (0.5, 3.5)	-	-
White	1.1 (0.4, 3.5)	-	-	-
On HAART	5.5 (1.4, 21.5)*	-	2.7 (0.9, 8.1)	-
Undetectable viral load	0.3 (0.1, 0.9)*	-	-	-

All the results were from multiple logistic regression analysis. Non-smoker group was the reference group in all the analysis. Reference group for other variables was non-marijuana user, non-white subject, subject who was not on HAART and subject who had detectable viral load respectively.

- The variable had p > 0.2 in uni-variate analysis and was not selected into multiple logistic regression analysis.

*p < 0.05 for the OR. ** p < 0.01 for the OR. *** p < 0.001 for the OR.

^a ^Subject having no cough was reference group. Nagelkerke R^2 ^= 0.252.

^b^Subject having no sputum was reference group. Nagelkerke R^2 ^= 0.177.

^c^Subject having no respiratory symptom at all was reference group. Respiratory symptom was defined as cough, sputum or breathlessness. Nagelkerke R^2 ^= 0.194.

^d ^Subject having either abnormal lung function or any respiratory symptom was reference group. Abnormal lung function was defined as either FEV_1_/FVC < 70% or %FEV_1_<80% or %FVC < 80%, either pre- or post-salbutamol test. Nagelkerke R^2 ^= 0.128.

#### Sputum

Fifty-one (43%) subjects produced sputum, including 11 (25%) non-smokers, 8 (35%) former smokers and 32 (63%) current smokers (p = 0.001) (Table 1). Compared to non-smokers, current smokers had higher odds of sputum, OR = 5.0 (95% CI: 1.9, 13.3) after controlling for marijuana use (p = 0.001). For former smokers the OR of 1.7 (95% CI: 0.5, 5.3) was not significant compared to non-smokers (p = 0.382). No interaction existed. The results are presented in Table 3.

#### Breathlessness

Eight subjects (7%) had breathlessness (Table 1). All of them were smokers including 1 former smoker and 7 current smokers (p = 0.027), however we could not compute the OR because of 0 cases in the reference (non-smoker) group, nor could we use logistic regression to estimate the risk factors further.

#### Any symptom

**Figure 1:**
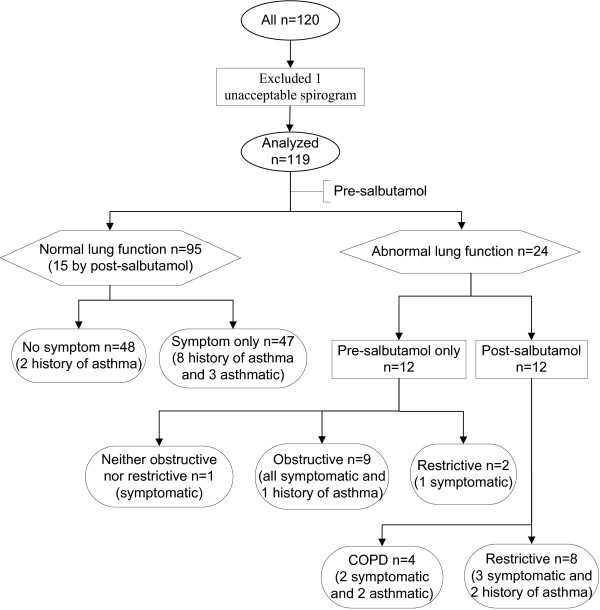
**Study flow chart and subject classifications**. Normal lung function was defined by FEV_1_/FVC ≥ 70% and %FEV_1 _≥ 80% and %FVC ≥ 80%, by both pre- and post-salbutamol tests. Pre-salbutamol values were used to classify normal lung function if post-salbutamol test was not done. Abnormal lung function was defined by either FEV_1_/FVC < 70% or %FEV_1 _< 80% or %FVC < 80%, either pre- or post-salbutamol test. Symptomatic was defined as having cough, sputum or breathlessness. Obstructive lung function was classified as pre-salbutamol FEV_1_/FVC < 70% without post-salbutamol values. COPD was defined as post-salbutamol FEV_1_/FVC < 70%. Restrictive lung function was defined by FEV_1_/FVC ≥ 70% and %FVC < 80%, either before or after salbutamol inhalation. Asthma was defined as reversible FEV_1_, which improved more than 12% and 200 ml after salbutamol inhalation.

In terms of three respiratory symptoms (cough, sputum and breathlessness), 63 (53%) subjects had at least one respiratory symptom, including 16 (36%) non-smokers, 9 (39%) former smokers and 38 (73%) current smokers (p = 0.001) (Table 1). Compared to non-smokers, current smokers were 4.9 (95% CI of OR: 2.0, 11.8) times more likely to have at least one symptom, after controlling for current HAART status (p < 0.001). For former smokers the OR was 1.0 (95% CI: 0.3, 2.8) compared to non-smokers (p = 0.969). No interaction existed. The results are presented in Table 3 and Figure 1.

### Association between respiratory diseases and smoking

#### COPD

Three (3%) subjects were diagnosed with COPD originally. In addition, one subject with borderline lung function was diagnosed with COPD by the committee. All these 4 cases of COPD were moderate in severity according to GOLD guidelines, and asthma co-existed in 2 COPD cases. All of them were white men, mean (SD) age was 49.8 (7.3), ranging from 43 to 57 years old. All were smokers with mean (SD) pack-years of 29.5 (13.8), however COPD was not associated with ever smoking status (p = 0.295), probably due to the small number of cases. The OR could not be calculated because of zero case in non-smokers. Notably 14 (12%) subjects had obstructive lung function by pre-salbutamol spirometry testing, however only 5 (38%) of them underwent post salbutamol testing. Therefore we could not confirm the other 9 potential COPD subjects. Among 14 subjects with pre-salbutamol obstructive lung function, there were 2 non-smokers, 3 former smokers and 9 current smokers. Although smokers had 4.0 times the odds of pre-salbutamol obstructive lung function, this crude OR was not significant (p = 0.079) and its 95% CI (0.9, 18.8) was very wide. COPD results are presented in Table 1 and Figure 1.

#### Restrictive lung function

Ten (8%) subjects had restrictive lung function. Mean (SD) age was 42.5 (7.0), ranged from 34 to 56 years old. Seven (70%) of them were women, including 6 black women. Amongst 10 subjects with restrictive lung function, there were 4 smokers including 1 former smoker and 3 current smokers (p = 0.170) (Table 1). Multiple logistic regression analysis was not conducted because there were ten cases of restrictive lung function only. Results are also presented in Figure 1.

#### Asthma

Thirteen (11%) subjects had a history of asthma, which was not associated with smoking status (p = 0.553) (Table 1). Only 5 (38%) of them underwent post-salbutamol testing and 2 subjects were asthmatic at the time of study. In addition, among 106 subjects without an asthma history, 3 (3%) were asthmatic. In total 5 subjects were asthmatic at the time of study, which was not associated with smoking status (p = 0.985). In total 16 (13%) subjects were asthmatic or had an asthma history, which was not associated with smoking status (p = 0.551). A history of asthma and spirometry diagnosed asthma are also presented in Figure 1.

#### Respiratory diseases history

A history of bronchitis was present in 38 (32%) subjects, pneumonia in 46 (39%) subjects, tuberculosis in 5 (4%) subjects, emphysema in 1 (1%) subject and asthma in 13 (11%) subjects. Former smokers had 3.7 times the odds of a history of bronchitis than non-smokers (95% CI: 1.1, 12.5), after controlling for race and HAART use (p = 0.038), while the OR of 1.5 (95% CI: 0.6, 4.1) for current smokers was insignificant (p = 0.421). Moreover, white subjects had 12.4 (95% CI: 1.5, 101.7) times the odds of a history of bronchitis compared to non-white subjects (p = 0.019). A history of other respiratory diseases was not associated with smoking status.

### Subject classifications and their association with smoking

The exclusive subject categories based on lung function, respiratory symptoms, COPD and restrictive lung disease are shown in the study flow chart (Figure 1). The category of 'normal lung function without symptom' represented subjects who had normal lung function and did not have any respiratory symptoms. Forty-eight (40%) subjects were in this category, including 2 subjects with a history of asthma. In this category there were 24 non-smokers, 12 ex-smokers and 12 current smokers, accounting for 55%, 52% and 23% in each smoking category respectively (p = 0.004) (Table 1). All potential confounders had p > 0.2 in uni-variate analysis except smoking status. Comparing to non-smokers, current smokers were 0.3 times less likely to be classified as 'normal lung function without symptom', OR = 0.3 (95% CI: 0.1, 0.6) (p = 0.002). The OR of 0.9 (95% CI: 0.3, 2.5) was not significant for former smokers (p = 0.853). Results are listed in Table 3 and Figure 1.

## Discussion

We did not find excessive decline of %FEV_1 _in HIV-positive subjects compared with published reference ranges for the general population. In our study ten pack-years %FEV_1 _change was -2.1% (95% CI: -3.6%, -0.6%). In a population-based cross-sectional study, the %FEV_1 _before salbutamol in 2050 white people decreased by 0.29% (95% CI: -0.33%, -0.25%) for every one pack-years increment [17], which would equal an %FEV_1 _change of -2.9% (95% CI -3.3%, -2.5%) per ten pack-years. The findings in our study are comparable to that in the general population. Similar results were reported in previous studies, where HIV-positive subjects had similar loss of lung function as their HIV-negative counterparts [13,14], which did not support the hypothesis that lung function decline is greater in the HIV-positive population.

We should keep in mind that our study was cross-sectional and the effect of smoking we found might not apply to a cohort study [16,29]. In a cross-sectional study, for every one pack-years of smoking increment FEV_1_decreased by 7.4 ml (95% CI: 6.4, 8.4) in a typical male (173 cm tall) and by 4.4 ml (95% CI: 3.2, 5.6) in a typical female (161 cm tall) respectively [16]. While in this same population after 6-year follow up, the longitudinal analysis showed that among smokers, for every one pack/day of cigarette smoking, the rate of FEV_1 _decrease was 12.6 ml/year (95% CI: 9.7, 15.5) for men and 7.2 ml/year (95% CI: 4.8, 9.6) for women [29]. Therefore we should not extrapolate the same coefficient of pack-years of smoking found in a cross-sectional study to a prospective cohort study. In other words, we could not predict an HIV-positive smoker would decrease %FEV_1 _by 2.1% if s/he continued smoking for another 10 pack-years.

In the multiple regression model for %FEV_1_, the coefficient of gender was not significant (p = 0.640), and the point estimate of coefficient of pack-years did not change regardless of adjustment for gender, suggesting that gender did not affect %FEV_1 _in this HIV positive population. Similar results were reported in a meta-regression analysis where eight large population-based cross-sectional studies were synthesized: neither gender nor race affected the association of cigarette smoking with lung function measured by residual FEV_1 _(observed - expected value) [18]. However, other population-based studies showed that smoking affected the annual decrease of FEV_1 _significantly more in males than in females [16,19,29]. Further study is needed to compare the result in our study to the general population.

Our study found that current smokers had significantly higher odds of cough and sputum than either non-smokers or former smokers, while the difference between non-smokers and former smokers was not significant, after controlling for possible confounders. The findings were consistent with other studies [8,30,31]. Therefore, effective smoking cessation projects would help HIV-positive smokers to have less cough and sputum. Moreover, the prevalence of smoking in our study was 2.4 (95% CI: 2.0, 3.0) times higher than that in Ontario general population in 2007 [2], which reinforced the need for smoking cessation programmes in the HIV-positive population. Fortunately 20 (38%) of current smokers were trying to quit smoking at the time of study. Fifteen (65%) former smokers quit smoking successfully without medication or counselling, implying insufficient involvement of health care providers in terms of helping smokers quit. Further, our study showed an association of smoking with childhood household smoking environment (p = 0.023): current smokers accounted for 7 (24%) of those subjects whose parents did not smoke, 12 (33%) if the father smoked, 6 (60%) if the mother smoked and 27 (63%) if both parents smoked. Therefore, an effective smoking cessation program should target not only current smokers, but also health professionals and families.

Marijuana use was evaluated in our study when the effect of smoking was estimated. Marijuana use might be associated with respiratory symptoms such as cough and sputum production. We found current marijuana users tended to use more frequently and for longer time than former users, however we did not know how many joints a subject consumed each time. More measurement of cumulative marijuana consumption might be more helpful to further examine the effect of marijuana use more deeply.

Subjects in our study represented the source population at the SIS clinic fairly well. Only five patients refused participating. In a clinical database of our study population (data unpublished), mean (SD) age among 726 active patients was 43.0 (10.5) years old in 2007, males accounted for 68% (95% CI: 65%, 72%), and smoking prevalence was 48% (95% CI: 44%, 53%). Compared to this clinical database, the subjects in our study was comparable in terms of age (p = 0.718) and smoking prevalence (p = 0.365), however we recruited a slightly greater proportion of males in our study (rate ratio RR = 1.1, 95% CI: 1.0, 1.3). Notably the smoking prevalence of 44% (95% CI: 35%, 53%) in our study was significantly lower than 58% (95% CI: 55%, 61%), the lowest smoking prevalence in OCS over time in 2007 (data unpublished). As OCS was a province-wide study, we considered it the best resource to assess smoking prevalence in Ontario HIV-positive population, although the subjects in OCS might not represent the whole HIV positive population in Ontario due to voluntary participation. Nevertheless the representativeness of our study subjects was limited to our clinic only.

Since we only detected four cases of COPD, we had low power to examine the effect of smoking on COPD. Nevertheless all four COPD cases were smokers, and smokers had a crude OR of 4.0 (95% CI: 0.9, 18.8) of pre-salbutamol obstructive lung function compared to non-smokers in our study. In a prospective observational study with 867 HIV-positive veterans, either former or current smokers were 5.3 times more likely to develop COPD than non-smokers (95% CI was 1.5 to 18.0 for former smokers and 1.6 to 17.0 for current smokers) [10]. Our study was comparable with these results, albeit underpowered to detect statistically significant difference due to the small number of COPD cases.

We likely would have captured more cases of COPD, if all the subjects with pre-salbutamol obstructive lung function had undergone post-salbutamol testing. According to our preset criteria, all subjects with %FEV_1 _< 90% should undergo post salbutamol test, which should have included 46 subjects. However only 27 (59%) of these subjects agreed to salbutamol inhalation followed by repeat spirometry, primarily due to time limitations. As a result, amongst 14 subjects whose spirogram suggested COPD by pre-salbutamol test, 9 (64%) subjects did not undergo post-salbutamol test and could not be confirmed. We might expect 5 to 6 more cases of COPD in our study. In a prospective observational study the prevalence of COPD in 1014 HIV positive veterans was 10% by ICD-9 codes and 15% by self report respectively [9]. Comparing this HIV-positive veteran population to our study population, the median age of study population was 50 versus 44.0 years old, the median age of COPD cases was 52 (by ICD-9 codes, 51 by self report) versus 49.5 years old. COPD usually is often diagnosed in patients 50 years or older, and longer follow up will be needed to observe development of additional COPD cases.

## Conclusions

In conclusion, we found cigarette smoking affected HIV infected subjects similarly to estimates of its effect in the general population. Cumulative cigarette consumption was associated with worse lung function and higher odds of respiratory symptoms. However the loss of %FEV_1 _did not accelerate in HIV-positive population compared to the general population. Current smokers were at significant higher odds to present respiratory symptoms compared to non-smokers, but former smokers were at the similar risk compared to non-smokers. Although all four COPD cases had smoked, we could not evaluate the effect of smoking on COPD due to small number of cases. More participants and longer follow up would be needed to estimate the effect of smoking on COPD development. Our study highlighted the importance of smoking cessation in the HIV-positive population in terms of improving lung function and reducing respiratory symptoms, and may prevent the development of COPD.

## Competing interests

Qu Cui and Marek Smieja are currently leading an open label study sponsored by the Pfizer company, where we offer Champix to HIV-positive smokers to help them quit smoking and we evaluate the effectiveness, safety and tolerability of Champix in this HIV-positive population.

## Authors' contributions

QC wrote study protocol, designed the questionnaire, carried out medical chart review, performed the statistical analysis and drafted the manuscript. SC performed the spirometry test, carried out the questionnaire survey and coordinated the study. AM made substantial contributions to interpretation of data, and was involved in revising the draft critically for important intellectual content. FS made substantial contributions to acquisition of data, and was involved in revising the draft critically for important intellectual content. LT made substantial contributions to analyze data, and was involved in revising the draft critically for important intellectual content. MS conceived of the study, participated in its design, made substantial contributions to acquisition of data, and helped to draft the manuscript. All authors read and approved the final manuscript.

## Authors' information

QC: PhD student in Health Research Methodology Program in Department of Clinical Epidemiology and Biostatistics at McMaster University.

SC: Respiratory technologist at St. Joseph's Healthcare.

AM: Professor in Department of Medicine (respirology) at McMaster University.

FS: Chair, professor in Department of Pathology and Molecular Medicine (microbiology) at McMaster University.

LT: Associate Professor in Department of Clinical Epidemiology and Biostatistics at McMaster University.

MS: Associate Professor in Department of Pathology and Molecular Medicine at McMaster University, and at St. Joseph's Healthcare, Hamilton.
